# The role of *MDM2* amplification and overexpression in therapeutic resistance of malignant tumors

**DOI:** 10.1186/s12935-019-0937-4

**Published:** 2019-08-22

**Authors:** Helei Hou, Dantong Sun, Xiaochun Zhang

**Affiliations:** Department of Medical Oncology, The Affiliated Hospital of Qingdao University, Qingdao University, 16 Jiangsu Road, Qingdao, 266005 China

**Keywords:** MDM2, p53, Therapeutic resistance, Molecular mechanism

## Abstract

The MDM2 protein encoded by the mouse double minute 2 (*MDM2*) gene is the primary negative regulatory factor of the p53 protein. MDM2 can ligate the p53 protein via its E3 ubiquitin ligase, and the ubiquitinated p53 can be transferred to the cytoplasm and degraded by proteasomes. Therefore, MDM2 can maintain the stability of p53 signaling pathway. *MDM2* amplification has been detected in many human malignancies, including lung cancer, colon cancer and other malignancies. MDM2 overexpression is associated with chemotherapeutic resistance in human malignancies. The mechanisms of chemotherapeutic resistance by MDM2 overexpression mainly include the p53–MDM2 loop-dependent and p53–MDM2 loop-independent pathways. But the role of MDM2 overexpression in tyrosine kinase inhibitors resistance remains to be further study. This paper reviews the possible mechanisms of therapeutic resistance of malignancies induced by *MDM2* amplification and overexpression, including chemotherapy, radiotherapy, targeted agents and hyperprogressive disease of immunotherapy. Besides, MDM2-targeted therapy may be a potential new strategy for treating advanced malignancies.

## Background

Malignancies are both a leading cause of death and a serious social and economic problem especially in China. Per the Cancer Country Profiles in China reported by the World Health Organization (WHO), the total number of cancer-related deaths in 2014 was 2,205,200 (including 1,425,700 men and 779,500 women), accounting for 22.40% of the total deaths in 2014 [1]. In addition to traditional treatment (surgery combined with radiotherapy and chemotherapy), tyrosine kinase inhibitors (TKIs) provides more therapeutic options for patients with malignancies. Epidermal growth factor receptor (EGFR) inhibitors, such as gefitinib and erlotinib, benefit advanced non-small cell lung cancer (NSCLC) patients harboring sensitive EGFR mutations [2]. The use of small molecular TKIs targeting other mutation sites of NSCLC, such as anaplastic lymphoma kinase (ALK), is also developing rapidly [3]. However, the serious problem with TKIs treatment is drug resistance. In addition to secondary drug resistance caused by heterogeneity of malignancies [4], primary drug resistance limits the TKIs treatment of malignancies [5].

**Fig. 1:**
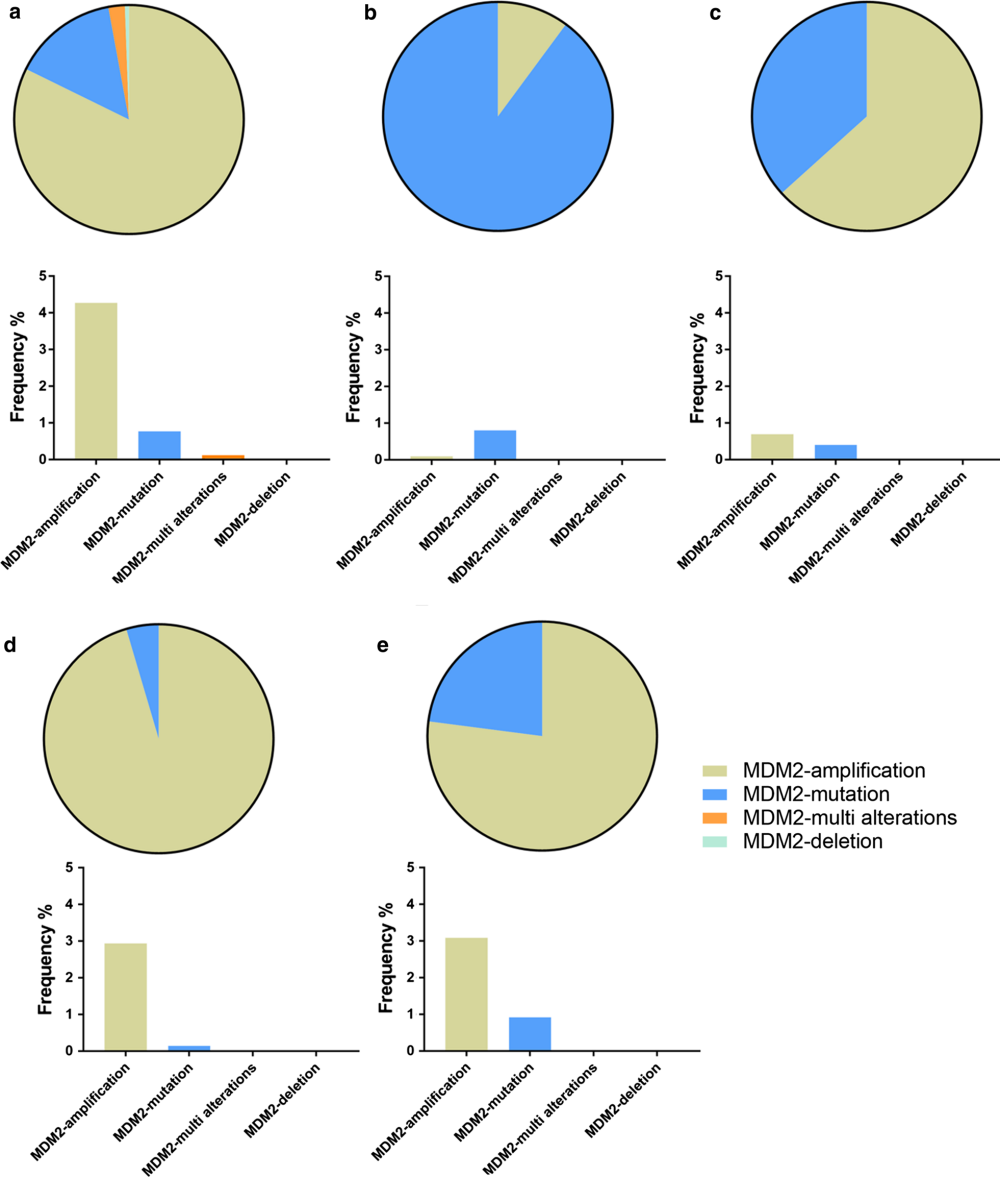
The types and frequencies of MDM2 alterations in different malignancies (cBioportal for Cancer Genomics). *A* lung cancer, *B* colorectal cancer, *C* livercancer, *D* breast cancer; *E* esophagogastric cancer

Murine double minute 2 (*MDM2*) is one of 3 highly amplified genes (*MDM1*, *MDM2* and *MDM3*) initially found in the Balb/c3T3 fibroblast cell line (3T3DM) of tumor-bearing mice [6–8]. However, only *MDM2* gene exists in the cellular genomes of human malignancies such as lung cancer and colon cancer [9]. The genomic datasets from the datasets of cBioportal for Cancer Genomics revealed that MDM2 overexpression can be detected in many malignancies like lung cancer, breast cancer, liver cancer, esophagogastric cancer and colorectal cancer. MDM2 overexpression is the most common alteration especially in lung cancer patients. The alterations and frequencies of *MDM2* in malignancies are concluded in Fig. 1. MDM2 overexpression has been found in both malignant tumor cells and patients with primary resistance to EGFR inhibitors. The paper reviews the relationship between *MDM2* overexpression and drug resistance, especially TKIs resistance, in treating malignancies.

## *MDM2* gene

The human *MDM2* gene is located on the long arm 13–14 of chromosome 12 (12q13-14). Olineret et al. [10] first discovered it in 1992 using an *MDM2* gene probe. The *MDM2* gene encodes a 483-amino acid protein with a molecular weight of approximately 90 kDa. The N-terminal of the MDM2 protein begins with region I, which is the MDM2–p53 binding region. Region I of MDM2 can bind to the p53 protein and can also bind to the gene promoter to activate the corresponding gene expression. This region is also involved in the binding process to other proteins. Region II is the central acidic region of MDM2 that binds to the ribosomal L5 protein. This region also mediates the specific binding of MDM2-L5 or MDM2-L5-p53 complexes with 5sRNA. Combining the latter two molecular complexes is a precursor of ribosomal assembly in mammalian cells. Region III is a zinc finger motif. Region IV is ring finger domain, which mediates the combination of protein–protein and protein–nucleic acids. This region also mediates the combination of sequence-specific ribonucleic acids (RNA) [11, 12].

## The biology function of MDM2

### The relationship between MDM2 and p53

MDM2 is a primary negative regulatory factor for p53. Under physiological conditions, close-loop negative feedback regulation of MDM2–p53 contributes to normal p53 protein expression. The expressions of MDM2 and p53 are low under this normal condition. MDM2 binds to p53 through its N-terminal domain (Region I) and forms the MDM2–p53 complex. The binding process obscures the p53 transcription activation region and reduces p53 transcription activity. The ring finger domain of MDM2 can then act as an E3 ligase and ubiquitinate p53. Proteasome interaction then leads to p53 protein degradation [13]. MDM2 can also directly remove p53 from cells to reduce p53 protein levels [11].

### The relationship between MDM2 and other proteins

The p21 protein is a downstream protein of the p53 signaling pathway, whose transcription is activated by p53. P21 is the key molecule that determines cell survival. The expression of p21 causes cell cycle arrest, promotes apoptosis and enhances chemotherapeutic sensitivity [14]. The combination of MDM2 and p21 promotes degradation of p21 mediated by proteasome interaction [15]. MDM2 can enhance p21 degradation by proteasome interaction and is involved in regulating p21 protein levels [16, 17]. In addition, MDM2 is associated with various protein molecules such as E2F, p19 (Arf) and Ras-mitogen-activated protein kinase (MAPK). This review mainly discusses the relationship between MDM2 and cancer treatment resistance, especially TKI resistance, therefore, the relationship between MDM2 and other protein molecules will not be restated.

## Role of MDM2 and therapeutic resistance

### MDM2 overexpression and chemotherapeutic resistance

#### MDM2–p53 loop-dependent drug resistance

In 1995, Kondoet et al. confirmed the possible relationship between MDM2 and chemotherapeutic resistance in malignancies. MDM2 is suggested to inhibit cisplatin-induced apoptosis, which may lead to cisplatin resistance in human malignancies [18]. Hayashiet et al. confirmed that the MDM2–p53 loop contributes to cisplatin resistance in epidermoid carcinoma. P53 protein phosphorylation induced by cisplatin can reduce the cisplatin resistance, while increased MDM2 expression and non-phosphorylated p53 can be detected in cisplatin-resistant tumor cells. This process may be related to the regulation of loop auxiliary factor p73. Enhancing factor p73 expression was found to enhance cisplatin resistance mediated by the MDM2–p53 loop in tumor cells [19]. Therefore, MDM2 overexpression plays an important role in the resistance to cisplatin. MDM2 overexpression itself can directly inhibit the sensitivity to cisplatin, at the same time, the down-regulation of p53 induced by MDM2 overexpression may lead to the resistance to cisplatin.

O_6_-methylguanine methyltransferase (MGMT) can demethylate temozolomide and induce temozolomide resistance in glioma tumor cells. MDM2–p53 negative feedback regulation pathway contributes to the mechanism of temozolomide resistance. The inhibition of MDM2 expression can up-regulate p53 expression and then down-regulate MGMT, while down-regulating MGMT expression enhanced tumor cells’ sensitivity to temozolomide [20].

MDM2 can inhibit the expression of wtp53, rendering tumor cells resistant to chemotherapeutic agents. In vitro experiments showed that breast cancer cells transfected with amplified *MDM2* were more likely to obtain drug resistance to doxorubicin. In addition, MDM2 expression in doxorubicin-resistant cell lines was significantly higher than that in doxorubicin-sensitive cell lines detected by immunohistochemistry (IHC). MDM2-induced doxorubicin resistance in tumor cells was mainly achieved by down-regulating wtp53 expression, while MDM2 had no this effect on mtp53 cell lines [21].

Sheng et al. also confirmed the important role of the MDM2–p53 loop in chemotherapeutic resistance. When treated with gemcitabine and cisplatin, nucleic acid damage induced by chemotherapeutic agents activates the expression of both MDM2 and wtp53. Meanwhile, another functional protein, musashi-2 (MSI-2), can enhance the E3 ligase function of MDM2 by continuously up-regulating MDM2 expression to accelerate wtp53 ubiquitination. Silencing MSI-2 can downregulate MDM2 and, at the same time, upregulate the expression of wtp53, which increases tumor cells’ sensitivity to gemcitabine and reduces the tumor cells’ invasive ability. This experiment confirmed the role of MDM2–p53 negative feedback loop in chemotherapeutic drug resistance in tumor cells and showed that MSI-2, as an important regulatory factor of the MDM2–p53 loop, plays an important role in drug resistance of tumor cells [22].

In addition, other protein molecules, such as homeobox A13 (HOXA13), play important roles in fluorouracil (5-FU) resistance in tumor cells by regulating the MDM2–p53 loop [23]. Therefore, MDM2–p53 negative-feedback loop is associated with the resistance in many malignancies to different chemotherapeutic drugs.

#### MDM2–p53 loop-independent drug resistance

In addition to the classic MDM2–p53 loop dependent chemotherapeutic resistance in tumor cells, MDM2 can achieve chemotherapeutic resistance through a p53-independent pathway. MDM2 can increase chemotherapeutic resistance by inducing the epithelial mesenchymal transition (EMT) process [24], which is not only related to chemotherapeutic resistance but to target agents.

### Relationship between MDM2 overexpression and radiotherapy

MDM2 may not only contribute to resistance to chemotherapy, but also confirmed to be associated with radiotherapy insensitivity of malignancies. Adenovirus-mediated p53 gene therapy can inhibit the binding of p53 and MDM2, which reduces p53 degradation and enhances tumor cells’ sensitivity to radiotherapy, especially tumor cells with MDM2 overexpression [25]. As a result, MDM2 overexpression may be a key factor in the insensitivity of tumor cells to radiotherapy. Feng et al. confirmed that treatment with the small molecular MDM2 inhibitor, MI-219, reduced p53 degradation and enhanced the sensitivity of tumor cells to radiotherapy through the p53–MDM2 loop [26].

In conclusion, MDM2 overexpression contributes to the resistance to traditional chemotherapy and radiotherapy via MDM2–p53 loop dependent pathway and EMT pathway which indicated the potential method for the detection of therapeutic resistance of malignancies and a novel treatment option. Besides, combination of MDM2 inhibitors and chemotherapy or radiotherapy may reverse of outcome of treatment and benefit patients.

### MDM2 overexpression and TKIs resistance

Few studies have focused on the relationship between MDM2 and TKIs in malignancies while some studies have shown that combination of MDM2 inhibitors and the corresponding TKIs have synergistic effects on cancer treatment. In vitro experiment of Bianco et al. demonstrated that the treatment of MDM2 anti-sense oligodeoxy-nucleotides combined with gefitinib, a small molecular EGFR TKI,significantly inhibited the proliferation of prostate cancer cells [27]. Furthermore, ALK inhibitors (crizotinib, ceritinib) combined with a MDM2 inhibitor, CGM097, were confirmed to be more effective than single agent of ALK TKI for neuroblastoma cell lines with ALK mutations and wtp53 [28]. Clinically, MDM2 was also associated with drug resistance in other targeted treatment of malignancies. A study confirmed that MDM2 overexpression is associated with trastuzumab resistance in human epidermal growth factor receptor-2 (HER-2) positive breast cancer [29]. Another recently clinical study revealed that MDM2 overexpression in NSCLC patients harboring EGFR sensitive mutation is associated with a poor progression free survival (PFS) [30]. But the mechanism of MDM2 overexpression in the resistance to TKIs remains unclear. As far as we know, there is no research on the relationship between MDM2 overexpression and TKIs resistance. Whether the treatment of TKIs combined with MDM2 inhibitor will benefit patient needs further study. The inferential molecular mechanisms from the latest studies on TKIs resistance caused by MDM2 overexpression are summarized below.

#### Signaling pathway bypass activation

The classic nuclear factor-κB (NF-κB) signaling pathway was confirmed to be a pivot of multiple signaling pathways and was shown to be associated with EGFR TKIs resistance in NSCLC patients [31, 32]. Normal expression of MDM2 has a dual effect on the NF-κB signaling pathway. For cells expressing normal levels of NF-κB, MDM2 causes excessive activation of NF-κB and promotes cell proliferation, while for cells with sustained overexpression of NF-κB, MDM2 inhibits the NF-κB signaling pathway and promotes apoptosis [33]. However, for cells with overexpression of MDM2, MDM2 overexpression can promote the expression of the NF-κB precursor, p100, through p53–MDM2 loop-dependent or loop-independent pathways and then activate the NF-κB signaling pathway. The activation of NF-κB signaling pathway can reduce tumor cell death and lead to drug resistance [34]. MDM2 can also act as a ubiquitin ligase to hydrolyze the EGFR phosphorylated peroxisome proliferator activated receptor-γ (PPAR-γ), resulting in the accumulation of the NF-κB/p65 protein and activation of the NF-κB signaling pathway [35]. These results suggest that MDM2 overexpression can activate the NF-κB signaling pathway to achieve the resistance to EGFR TKIs in malignancies via signaling pathway bypass activation.

#### Inhibition of tumor cell apoptosis

Bcl-2 family proteins contribute to the balance of cell apoptosis. The Bcl-2 family members are divided into apoptotic molecules, such as Bax, and anti-apoptotic molecules such as Bcl-2 and Bcl-XL. Upregulating Bcl-2 expression and imbalance expression of Bcl-2 family proteins had been confirmed to be closely related to EGFR TKIs resistance in NSCLC [36]. Several studies confirmed that after inhibiting MDM2 expression, the expression of p53 and Bax can be up-regulated, while Bcl-2 expression can be down-regulated, resulting in increased drug sensitivity in tumor cells [37, 38]. Therefore, changing the Bcl-2 family balance could be one mechanism of TKIs resistance induced by MDM2 overexpression in malignancies.

#### Promotion of epithelial mesenchymal transformation

EMT process is associated with drug resistance in tumor cells via enhancing the migration ability of tumor cells. Stromal phenotypic tumor cells obtain stem cell-like characteristics and enhance their drug resistance through EMT [39]. MDM2 overexpression can promote EMT process in tumor cells to achieve chemotherapeutic drug resistance [24]. The EMT process is also associated with TKIs resistance in malignancies. Studies by Weng et al. confirmed that the EMT process is associated with gefitinib (1st generation EGFR TKI) and osimertinib (3rd generation EGFR TKI) resistance in NSCLC patients harboring EGFR sensitive mutations [40]. Studies have also confirmed that drug resistance to ALK TKIs in ALK positive NSCLC patients may be associated with the EMT process [39, 41]. Given that MDM2 overexpression can act as a strongly promoter of EMT process, MDM2 overexpression is likely to promote the EMT process to achieve TKIs resistance in malignancies.

#### Promotion of sustained angiogenesis

**Fig. 2:**
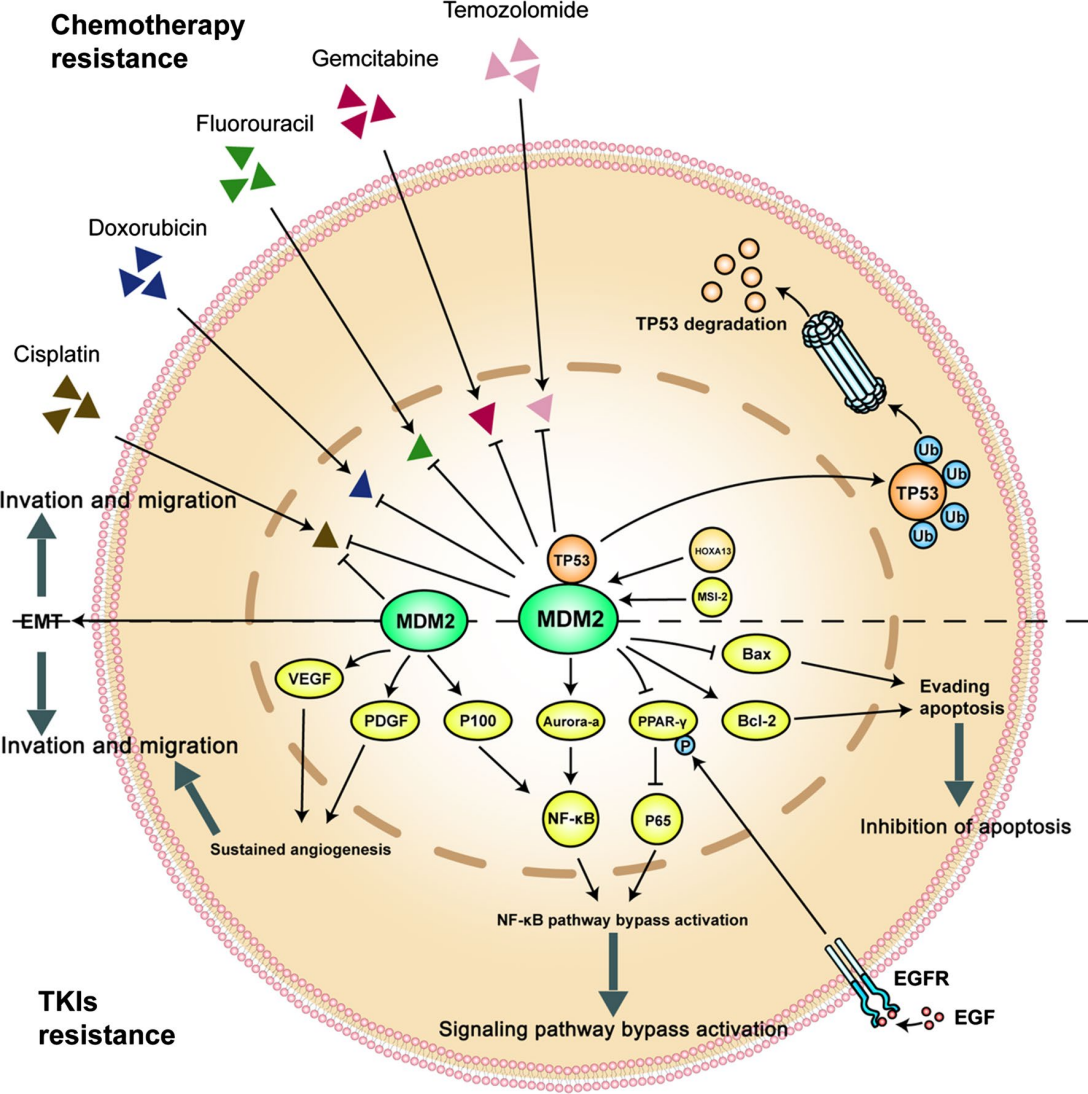
The role of MDM2 in chemotherapy and TKIs resistance

Tumor angiogenesis enables tumor cells to infiltrate the stroma and metastasize to distant region. As early as in 1996, studies using immunohistochemistry (IHC) confirmed that high expression of vascular endothelial growth factor (VEGF) and platelet-derived endothelial growth factor (PDGF) were detected in MDM2 overexpressed breast cancer cells, suggesting that MDM2 may be involved in angiogenesis process of maliganancies [42]. Similarly, recent studies demonstrated that MDM2 overexpression promotes angiogenesis process by inducing the balance of cytokine expression into an angiogenic status to achieve angiogenesis and enhance the migrative and invasive ability of tumor cells [43]. The combination treatment of the VEGF inhibitor, bevacizumab, and the MDM2 inhibitor, nutlin-3, significantly inhibits tumor proliferation and angiogenesis [44]. Tumor angiogenesis is confirmed to be involved in drug resistance in tumor cells [45]. Therefore, MDM2 may be involved in TKIs resistance by promoting tumor angiogenesis although this mechanism has not been directly verified and requires further studies. The role of MDM2 overexpression in chemotherapy and TKI resistance was shown in Fig. 2.

According to the above findings, MDM2 seems to be the pivot of several cancerous biological behaviors including cell proliferation, apoptosis, tumor angiogenesis and metastasis which were verified to be associated not only with drug resistance to target therapy but other anti-cancer therapies. It indicated that MDM2 is likely to play an important role in the generation and development of cancer and may promote the resistance to targeted therapy and other anti-cancer therapies.

### MDM2 overexpression and hyperprogressive disease of immunotherapy

At present, cancer immune checkpoint inhibitors (ICIs) are wildly used for advanced cancer treatment. The PD-1 inhibitors, pembrolizumab and nivolumab, provide new treatment methods for advanced cancer patients. Advanced cancer patients with MDM2 overexpression developed tumor progression or even hyperprogressive disease after PD-1 inhibitor treatment [46]. In addition to the EMT and angiogenesis process induced by MDM2 overexpression in tumor cells, Zou et al. confirmed that MDM2 can act as an E3 ubiquitin ligase and reduce T cell activation by degrading transcription factor NFATc2 [47], resulting in the resistance to PD-1 inhibitors of malignancies. Besides, immunological tolerance may also participate in the hyperprogressive disease induced by MDM2 overexpression given that MDM2 served as a tumor associated antigen (TAA) in malignancies [48–50]. The role of MDM2 overexpression in the resistance to ICIs, especially the hyperprogressive disease after the treatment of PD-1 inhibitors, requires further studies. The inferential mechanisms of the resistance to all cancer therapeutics induced by MDM2 overexpression were summarized in Table 1.

**Table 1:** The mechanisms of therapeutics resistance induced by MDM2 amplification and overexpression

Alteration induced by *MDM2* amplification and overexpression	Downstream factors	Related therapeutic resistance	References
Inhibition of cisplatin-induced apoptosis	–	Cisplatin	[18]
MDM2–p53 loop	P53↓	Cisplatin, doxorubicin, gemcitabine, 5-FU, radiotherapy	[19, 21, 23, 25, 26]
MDM2–p53 loop	P53↓, MGMT↑	Temozolomide	[20]
EMT	Invasion and migration	Chemotherapy, target therapy	[24, 39–41]
MDM2–p53 loop	NF-κB signaling pathway↑	TKIs	[31–35]
MDM2–p53 loop	P53↓, Bax↓, Bcl-2↑	TKIs	[36–38]
MDM2–p53 loop	VEGF↑, PDGF↑	TKIs	[42–45]
NFATc2↓	T cell activation↓	Immunotherapy	[47]
Serves as a TAA	Immunological tolerance	Immunotherapy	[48–50]

## A novel therapy for cancer treatment by targeting MDM2

Preclinical and clinical evidences of MDM2 inhibitors for human cancer treatment had been proved that targeting MDM2 treatment can be a new method to improve efficacy and safety of cancer therapeutics [51]. Small molecule MDM2 inhibitors can block the binding of MDM2 and p53 by competitive binding to the region I of MDM2 protein, resulting in the increasing of p53 level and activation of p53 signaling pathway [52, 53]. As a result, small molecule MDM2 inhibitors can block the cell cycle, inhibit cell growth and promote cell apoptosis [54]. Nutlin-3 is a widely studied small molecular inhibitor of MDM2. Mechanisms of nutlin-3′s anti-tumor effect included direct cytotoxicity and retardation of proliferation by altering the tumor stroma and blood vessels in tumor microenvironment [55]. Since the appearance of nutlin-3, studies on small molecular MDM2 inhibitors have developed rapidly. For example, RG7112, a new compound developed based on nutlin-3, has four times more inhibitory effects on MDM2 than nutlin-3 [56]. Clinical trials of various small molecular MDM2 inhibitors are under way. Current studies suggest that for cancer patients with MDM2 overexpression, the treatment of MDM2 inhibitors combined with chemotherapy or corresponding targeted therapy was better for the improvement of efficacy and can reduce the drug resistance of malignancies [28, 57].

## Conclusion

The continuous development of anti-tumor drugs provides more options for advanced cancer patients. However, drug resistance has been a serious factor puzzling clinical treatment. The research on p53 signaling pathway have led to the discovery of possible therapeutic targets in this pathway. Among them, MDM2 is one of the most important target for cancer treatment. MDM2 overexpression plays an important role in the generation, development and drug resistance of malignancies. *MDM2* amplification and overexpression are involved in chemotherapy and radiotherapy resistance in tumor cells via clearly mechanisms. Besides, the role of MDM2 overexpression in TKIs resistance and hyperprogressive disease after immunotherapy requires further studies. In conclusion, *MDM2* amplification and overexpression play a great role in therapeutic resistance and targeting MDM2 might be a novel therapeutic strategy for cancer treatment.

## Data Availability

The data and material in this review all come from published papers.

## Abbreviations

EGFR: epidermal growth factor receptor
EGF: epidermal growth factor
MDM2: mouse double minute 2
TKI: tyrosine kinase inhibitor
MSI-2: musashi-2
OXA13: homeobox A13
NF-κB: nuclear factor-κB
PPAR-γ: peroxisome proliferator-activated receptor-γ
EMT: epithelial mesenchymal transition
TAA: tumor associated antigen
IHC: immunohistochemistry
ICI: immune checkpoint inhibitor
